# Enhancing Anchor Location Estimation Algorithm via Multi-Source Observations and Adaptive Optimization for UVIO

**DOI:** 10.3390/s26010019

**Published:** 2025-12-19

**Authors:** Boya Zhang, Gongliu Yang, Jin Wang, Guodong Lu

**Affiliations:** 1 School of Instrument Science and Opto-Electronics Engineering, Beihang University, Beijing 100191, China; zhangby512@buaa.edu.cn; 2 School of Mechanical Engineering, Zhejiang University, Hangzhou 310027, China; 3 The State Key Laboratory of Fluid Power and Mechatronic Systems, School of Mechanical Engineering, Zhejiang University, Hangzhou 310027, China; lugd@zju.edu.cn; 4 The Engineering Research Center for Design Engineering and Digital Twin of Zhejiang Province, School of Mechanical Engineering, Zhejiang University, Hangzhou 310027, China; 5 The Robotics Institute of Zhejiang University, Hangzhou 310027, China

**Keywords:** location, small curvature, LS-VCE, multi-observations, ultra-wide band, Unmanned Ground Vehicle

## Abstract

At present, the UWB-assisted VIO scheme only uses range measurements to estimate the anchor position. The accuracy of the anchor location estimation algorithm can be affected by factors such as the trajectory being a straight line or having a small curvature, as well as changes in multi-observation noise. To address these problems, we propose an adaptive UWB anchor location estimation algorithm leveraging Unmanned Ground Vehicle (UGV) multi-source observations. The key innovations include the following: (1) a novel anchor initialization method that incorporates both distance and angles, including azimuth and elevation measurements, to overcome the limitation of the approach that relies solely on range for straight or small-curvature trajectories; (2) an adaptive nonlinear optimization anchor location estimation algorithm that dynamically adjusts measurement weights and addresses the accuracy decreasing under time-varying noise characteristics in both distance and angle measurements caused by environmental disturbances. In this paper, the robustness and anchor position estimation accuracy of the proposed algorithm are validated through simulation and UGV real experiments.

## 1. Introduction

Accurate, reliable, and consistent localization remains a research problem to be solved for many robotic applications. In recent years, visual-inertial odometry (VIO) or visual-inertial simultaneous localization and mapping (VI-SLAM) have become popular solutions, such as [1,2,3], which enable accurate, high-rate attitude and velocity estimation, but sensor noise and computational errors cause the system to drift cumulatively over time. A common solution to reduce VIO cumulative drift is to add a global navigation system sensor, such that the positioning accuracy is stabilized within a certain range of error, as in a global navigation satellite system (GNSS) [4,5].

For indoor, tunnel, and corridor scenes, the GNSS signal is weak and cannot be used, and these scenarios may have factors that lead to the failure of visual feature matching, such as low light, similar textures in visual and geometric environments, and occlusions. Unlike GNSS and visual navigation, ultra-wideband (UWB) is one of the most recent and promising solutions in terms of accuracy, coverage range, and deployment cost [6] in the above scenarios. Ref. [7] introduced the time-of-flight (ToF) localization scheme based on UWB signals, which required at least four anchors for 3D localization. Range-based localization requires multiple known UWB anchors, which can increase cost and inhibit applicability in many spatially constrained scenarios [8,9,10,11].

More recently, approaches that employ only a single UWB anchor with an unknown position have been introduced [12,13,14,15]. Results indicate that by tightly coupling the UWB, camera, and Inertial Measurement Unit (IMU) measurements in a joint optimization problem, more accurate and robust localization can be achieved. This UWB-assisted VIO scheme only uses range measurement. For UWB anchors with unknown positions, relying solely on range measurements to achieve an accurate UWB anchor position estimation has certain requirements for the motion trajectory. It is difficult to obtain accurate anchor position results when moving in the same direction or only in curves with small curvature. In addition, the stochastic models of UWB range and angle measurements are not reliable sometimes. If fixed weights are used, it will affect the accuracy of the estimation algorithm. Therefore, the weights of different measurements need to be adjusted appropriately. To address these issues, in this paper, we propose an adaptive anchor location estimation algorithm integrating a multiple observations model and the least-squares VCE (LS-VCE).

## 2. Related Works

Most relevant to this paper is the work on using camera–IMU–UWB sensors for localization and mapping tasks. Some works employ VIO for on-board localization and use UWB ranging information to achieve relative localization between carriers [16,17,18,19]. Recent studies have shown that by fusing vision, inertial, and UWB data, it is possible to achieve the estimation of the unknown anchor position; at the same time, there exists a certain enhancement of the pose estimation.

Considering that the initial position of the UWB anchor is usually unavailable, Karol Hausman [15] proposed a multi-sensor fusion framework based on the Extended Kalman Filter (EKF) to estimate anchors and unmanned aerial vehicle (UAV) positions jointly. Xie et al. [20] proposed a loosely coupled approach to fuse a monocular camera with UWB ranging to a single anchor with an initial guess for its location to correct the scale problems of monocular visual odometry (VO). Wang et al. [21] proposed the First Estimation Jacobian Visual Inertial Ranging (FEJ-VIRO) using a long and short window structure to initialize the position of the UWB anchor, as well as the covariance used for state enhancement. The above approach only explores how to combine the camera, IMU, and UWB ranging when there is only one unknown anchor in the scene. A novel surface-based particle filter is proposed to localize the UWB anchors in the local world frame efficiently [22], which is also based on range measurement. The literature [23] compared the anchor position estimation performance of Least Squares Method (LSM), Weighted Least Least-Squares Method (LSM), Squares Method (WLSM), Particle Swarm Optimization (PSO) and weighted Particle Swarm Optimization (WPSO) for straight line trajectory, and achieved the result that when the trajectory shape is straight, none of the four methods can locate the position robustly and there is an inherent ambiguity in the range measurements for anchor positioning with a straight trajectory. Therefore, developing and researching a new precise anchor positioning method is particularly important.

Though the estimation accuracy by the optimized model will be improved, there exists a limitation. If the observation covariance is inaccurate, it may lead to unreasonable relative weight ratios between various types of observations during adjustment, thus adversely affecting the parameter estimation and the precision evaluation for adjustment results [24]. At present, there exist various variance component estimators (VCE) methods to solve the problems mentioned above [25,26,27,28,29,30]. These methods differ in the estimation principle employed, as well as in the distributive assumptions that need to be made. Among them, LS-VCE can be regarded as the general case of several other VCE methods. When the observation errors obey the normal distribution, the results obtained from LS-VCE are equivalent to those from other VCE methods.

The main contributions of this paper are as follows:

(1) Compared with existing distance-based anchor position estimation methods, the nonlinear optimization algorithm based on multiple observations used in this paper can still ensure the accuracy of the anchor estimation algorithm even when the motion trajectory is a straight line or has a small curvature.
(2) In order to balance weight ratios between various types of observations accurately, we integrate the LS-VCE algorithm into the above model to construct an adaptive nonlinear optimization anchor position estimation algorithm.
(3) The consistency and accuracy of the algorithm proposed in this paper are verified through simulation experiments and real UGV experiments, which improved the UWB anchor position estimation accuracy.

## 3. Measurement Model

**Figure 1:**
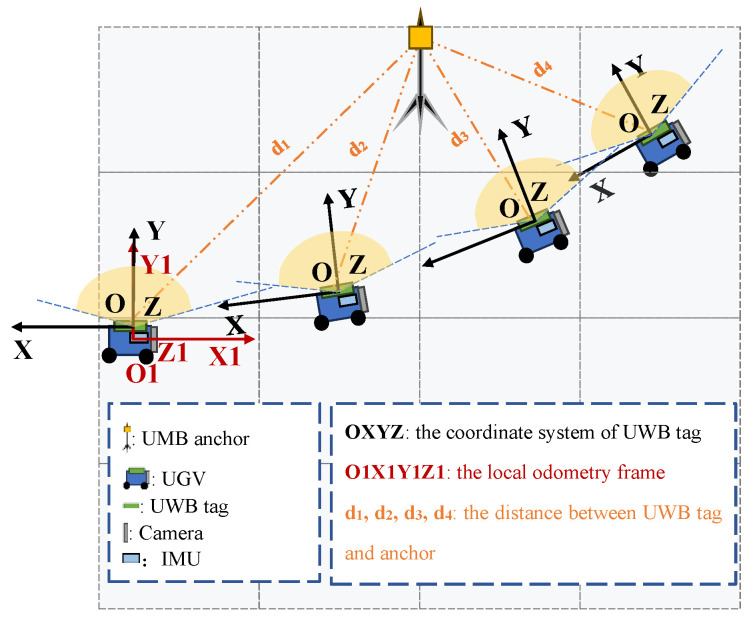
An illustration of the localization scenario.

Assume that multiple UWB anchors and a UGV are placed in a scene, and the location of the UWB anchor is unknown. As shown in Figure 1, it depicts the coordinate frames used in this work. Let $O_{1}-X_{1}Y_{1}Z_{1}$ represents the local odometry frame $\left\{C\right\}$, let O−XYZ represent the UWB tag coordinate system $\left\{U\right\}$.

The position of UGV in the local odometry frame $\left\{C\right\}$ is represented as $P^{C}={\left[\begin{array}{ccc} x_{1} & y_{1} & z_{1} \end{array}\right]}^{T}$, the position of UGV in the UWB tag coordinate system $\left\{U\right\}$ is represented as $P^{U}={\left[\begin{array}{ccc} x & y & z \end{array}\right]}^{T}$. The transformations between the two coordinate systems mentioned above are as follows:

(1) $$\left[\begin{array}{c} x\\ y\\ z\\ 1 \end{array}\right]=T\left[\begin{array}{c} x_{1}\\ y_{1}\\ z_{1}\\ 1 \end{array}\right],T=\left[\begin{array}{cc} R & p\\ 0 & 1 \end{array}\right]$$

(2) $$\left[\begin{array}{c} x_{1}\\ y_{1}\\ z_{1}\\ 1 \end{array}\right]=\left[\begin{array}{cc} R^{-1} & -R^{-1}p\\ 0 & 1 \end{array}\right]\left[\begin{array}{c} x\\ y\\ z\\ 1 \end{array}\right]$$

The homogeneous transformation matrix $T\in SE\left(3\right)$, which consists of the rotation matrix $R\in SO\left(3\right)$ and translation vector $p\in R^{3}$, represents the transformation from UWB tag coordinate system to local odometry frame. The rigid body transformation parameter between UWB tag coordinate system and local odometry frame is obtained through offline calibration.

This paper uses UWB technology for observation, including range d, azimuth α and elevation angle β, expressed as follows:

(3) $$d_{i}=\left\|P_{i}^{U}-P_{a}^{U}\right\|+\varepsilon_{d_{i}}$$

(4) $$\alpha_{i}=\arctan\left(y_{i}^{U}-y_{a}^{U},x_{i}^{U}-x_{a}^{U}\right)+\varepsilon_{\alpha_{i}}$$

(5) $$\beta_{i}=\arctan\left(\frac{z_{i}^{U}-z_{a}^{U}}{s_{i}}\right)+\varepsilon_{\beta_{i}}$$

With $s_{i}=\sqrt{{\left(y_{i}^{U}-y_{a}^{U}\right)}^{2}+{\left(x_{i}^{U}-x_{a}^{U}\right)}^{2}}$ representing the horizontal distance between the UGV and anchor, $P_{i}^{U}=\left[\begin{array}{ccc} x_{i}^{U} & y_{i}^{U} & z_{i}^{U} \end{array}\right]$ is the UGV position at time i in the UWB tag coordinate system. $P_{a}^{U}=\left[\begin{array}{ccc} x_{a}^{U} & y_{a}^{U} & z_{a}^{U} \end{array}\right]$ is the UWB anchor position in the UWB tag coordinate system. $\varepsilon_{d_{i}},\varepsilon_{\alpha_{i}},\varepsilon_{\beta_{i}}$ are range error, azimuth error and elevation angle error, respectively, which are assumed to follow a Gaussian distribution with zero mean and known standard deviation $\Omega_{d},\Omega_{\alpha },\Omega_{\beta }$.

## 4. Proposed Optimization Model

Previous reports estimated anchor position based on distance, which cannot guarantee estimation accuracy when the trajectory is a straight line or a small-curvature curve. For improving the accuracy of the UWB anchor position estimation algorithm using only distance measurement, we have established an optimization model for the unknown UWB anchor position estimation based on range/azimuth and range/azimuth/elevation angle.

### 4.1. Nonlinear Optimization Model and Solution Algorithm

The UWB range/azimuth/elevation angle residuals are notated as $e_{d}^{i},\;e_{\alpha }^{i},\;e_{\beta }^{i}$, respectively. They are defined as follows:

(6) $$\begin{array}{l} e_{d}^{i}\left(x_{a}^{U},y_{a}^{U},z_{a}^{U}\right)=d_{i}-\sqrt{{\left(x_{i}^{U}-x_{a}^{U}\right)}^{2}+{\left(y_{i}^{U}-y_{a}^{U}\right)}^{2}+{\left(z_{i}^{U}-z_{a}^{U}\right)}^{2}}\\ \left(i=1,2,3,\ldots ,k\right) \end{array}$$

(7) $$e_{\alpha }^{i}\left(x_{a}^{U},y_{a}^{U},z_{a}^{U}\right)=\alpha_{i}-\arctan\left(\frac{y_{i}^{U}-y_{a}^{U}}{x_{i}^{U}-x_{a}^{U}}\right)\left(i=1,2,3,\ldots ,k\right)$$

(8) $$e_{\beta }^{i}\left(x_{a}^{U},y_{a}^{U},z_{a}^{U}\right)=\beta_{i}-\arctan\left(\frac{z_{i}^{U}-z_{a}^{U}}{\sqrt{{\left(x_{i}^{U}-x_{a}^{U}\right)}^{2}+{\left(y_{i}^{U}-y_{a}^{U}\right)}^{2}}}\right)\left(i=1,2,3,\ldots ,k\right)$$

In Equations (6)–(8), $x_{a}^{U},y_{a}^{U},z_{a}^{U}$ represents the unknown position of the UWB anchor, $d_{i},\alpha_{i},\beta_{i}$ represents the distance measurement, azimuth angle, and elevation angle, respectively.

The cost function of m1 to be minimized is

(9) $$\underset{\left(x_{a}^{U},y_{a}^{U},z_{a}^{U}\right)}{\min}\left\{\sum_{i\in Ι}\rho \left({\left\|e_{d}^{i}\right\|}_{\Omega_{d}}\right)\right\}$$

The cost function of m2 to be minimized is

(10) $$\underset{\left(x_{a}^{U},y_{a}^{U},z_{a}^{U}\right)}{\min}\left\{\sum_{i\in Ι}\rho \left({\left\|e_{d}^{i}\right\|}_{\Omega_{d}}\right)+\sum_{i\in Ι}\rho \left({\left\|e_{\alpha }^{i}\right\|}_{\Omega_{\alpha }}\right)\right\}$$

The cost function of m3 to be minimized is

(11) $$\underset{\left(x_{a}^{U},y_{a}^{U},z_{a}^{U}\right)}{\min}\left\{\sum_{i\in Ι}\rho \left({\left\|e_{d}^{i}\right\|}_{\Omega_{d}}\right)+\sum_{i\in Ι}\rho \left({\left\|e_{\alpha }^{i}\right\|}_{\Omega_{\alpha }}\right)+\sum_{i\in Ι}\rho \left({\left\|e_{\beta }^{i}\right\|}_{\Omega_{\beta }}\right)\right\}$$

where m1 is the method that only uses range observations to estimate anchor position, m2 is the method that uses range and azimuth angle observations to estimate anchor position, and m3 represents the method that uses range, azimuth, and elevation angle observations to estimate anchor position. ρ is denoted as the Huber loss.

Equations (9)–(11) can be optimized using the standard Levenberg–Marquardt algorithm [5] with the Ceres solver [31]. The Jacobian matrix required for the Levenberg–Marquardt algorithm iteration steps is as follows:

(12) $$\begin{array}{l} \delta_{1i}={\left(z_{i}^{U}-z_{a}^{U}\right)}^{2}+{\left(x_{i}^{U}-x_{a}^{U}\right)}^{2}+{\left(y_{i}^{U}-y_{a}^{U}\right)}^{2}\\ \delta_{2i}={\left(x_{i}^{U}-x_{a}^{U}\right)}^{2}+{\left(y_{i}^{U}-y_{a}^{U}\right)}^{2}\\ J_{e_{d}^{i}\left(x_{a}^{U},y_{a}^{U},z_{a}^{U}\right)}=\left[\begin{array}{ccc} \frac{\left(x_{i}^{U}-x_{a}^{U}\right)}{\sqrt{\delta_{1i}}} & \frac{\left(y_{i}^{U}-y_{a}^{U}\right)}{\sqrt{\delta_{1i}}} & \frac{\left(z_{i}^{U}-z_{a}^{U}\right)}{\sqrt{\delta_{1i}}} \end{array}\right] \end{array}$$

$$J_{e_{\alpha }^{i}\left(x_{a}^{U},y_{a}^{U},z_{a}^{U}\right)}=\left[\begin{array}{ccc} \frac{-\left(y_{i}^{U}-y_{a}^{U}\right)}{\delta_{2i}} & \frac{\left(x_{i}^{U}-x_{a}^{U}\right)}{\delta_{2i}} & 0 \end{array}\right]$$

$$J_{e_{\beta }^{i}\left(x_{a}^{U},y_{a}^{U},z_{a}^{U}\right)}=\left[\begin{array}{ccc} \frac{-\left(z_{i}^{U}-z_{a}^{U}\right)\left(x_{i}^{U}-x_{a}^{U}\right)}{\delta_{1i}}\cdot \frac{1}{\sqrt{\delta_{2i}}} & \frac{-\left(z_{i}^{U}-z_{a}^{U}\right)\left(y_{i}^{U}-y_{a}^{U}\right)}{\delta_{1i}}\cdot \frac{1}{\sqrt{\delta_{2i}}} & \frac{\sqrt{\delta_{2i}}}{\delta_{1i}} \end{array}\right]$$

Since the UWB estimation method is performed online, a termination criterion is introduced to determine the uncertainty of the solution as [32], which uses the maximum singular value of the covariance matrix as a convergence judgment criterion of the UWB anchor estimation method.

### 4.2. Application of the LS-VCE for Optimization Model

The application of the LS-VCE for the optimization model aims to further enhance the estimation accuracy. The linear Gauss–Markov model can be written as:

(13) $$L=AX+e,E\left(L\right)=AX,D\left(L\right)=Q_{L}$$

where L is the m×1 observation vector influenced by random error vector e. X is the n×1 parameter vector that needs to be estimated. A denotes the m×n coefficient matrix and $Rank\left(A\right)=n<m$. $Q_{L}$ represents the m×m covariance matrix of e. When the covariance matrix $Q_{L}$ is not completely known to us, it can be expressed in the following form:

(14) $$Q_{L}=Q_{0}+\sum_{k=1}^{p}\sigma_{k}Q_{Lk}$$

where $Q_{0}$ is the known part of the covariance matrix, and in most cases, it is a null matrix. $Q_{Lk},k=1,2,\cdots ,p$ denotes the m×m prior cofactor matrices which are independent of each other. $\sigma_{k},k=1,2,\cdots ,p$ are the corresponding variance or covariance components that need to be estimated.

In the LS-VCE method, the least squares solution of various types of variance components can be obtained using the following formula:

(15) $$\hat{\sigma }=N^{-1}l$$

where $\hat{\sigma }$ denotes the vector composed of the estimated variance components (i.e., $\hat{\sigma }={\left[\begin{array}{cccc} {\hat{\sigma }}_{1} & {\hat{\sigma }}_{2} & \cdots & {\hat{\sigma }}_{p} \end{array}\right]}^{T}$). N denotes a p×p invertible normal matrix and l represents a column vector with dimension of p×1. The elements in N and l are calculated using the following two equations:

(16) nij=12trQLiQLPA⊥−1QLjQLPA⊥−1

(17) $$l_{i}=\frac{1}{2}{\hat{e}}^{T}Q_{L}^{-1}Q_{Li}Q_{L}^{-1}\hat{e}-\frac{1}{2}tr\left(Q_{0}Q_{L}^{-1}P_{A}^{⊥}Q_{Li}Q_{L}^{-1}P_{A}^{⊥}\right)$$

where i,j=1,2,⋯,p. $\hat{e}=P_{A}^{⊥}L$ represents the least squares residuals, in which $P_{A}^{⊥}$ is an m×m orthogonal projector, and $P_{A}^{⊥}=I-A{\left(A^{T}Q_{L}^{-1}A\right)}^{-1}A^{T}Q_{L}^{-1}$. $tr\left(\cdot \right)$ is the trace of a matrix. The specific derivation process can be found in Teunissen and Amiri-Simkooei [29]. Since both the covariance matrix $Q_{L}$ and the orthogonal projection $P_{A}^{⊥}$ are functions of the unknown variance components, the iteration calculation should be performed to obtain the final estimates.

To solve the issue of nonnegative estimation of the variance components, the inequality constraint σ>0 should be considered in the process of the LS-VCE method. When this constraint is included, the LS-VCE becomes an inequality-constrained least squares problem, which can further be converted into a quadratic programming (QP) problem as below [29]:

(18) $$\hat{\sigma }=\arg\underset{\sigma \geq 0}{\min}\left(\frac{1}{2}\sigma^{T}N\sigma -l^{T}\sigma \right)$$

In which the matrix N and the vector l are given in Equations (16) and (17).

To avoid the appearance of the singular covariance matrix that will affect the normal process of the adaptive anchor estimation algorithm, it should be pointed out that the inequality constraint σ>0 is replaced by $\sigma >c_{0}h$ ($c_{0}$ is a small positive number, e.g., 1.0 × E^−10^~1.0 × E^−12^; h is a p-D column vector in which each element is 1). The detailed steps of the proposed algorithm are as Algorithm 1.
**Algorithm 1.** Implementation of the Adaptive Nonlinear Optimization Anchor Position Estimation Method●Set the convergence thresholds of the iteration $\varepsilon_{0},\varepsilon_{1},\varepsilon_{2}$●Set the initial value of $P_{a}^{U}=\left[\begin{array}{ccc} x_{a}^{U} & y_{a}^{U} & z_{a}^{U} \end{array}\right]$;●Initialize $\hat{\sigma }={\left[\begin{array}{cccc} {\hat{\sigma }}_{1} & {\hat{\sigma }}_{2} & \cdots & {\hat{\sigma }}_{p} \end{array}\right]}^{T}$;Set: $Q_{Lk},k=1,2,\cdots ,p$;Calculate: ${\bar{Q}}_{L}=\sum_{k=1}^{p}{\hat{\sigma }}_{k}{\bar{Q}}_{Lk}$;●Set iteration count: i=0●Do for i●Set iteration count: j=0 and let $P_{a\left[0\right]}^{U}=P_{0}$;●Set auxiliary parameter ν=2 and the initial damping factor $\mu =\mu_{0}\max\left(diag\left(J^{T}{\bar{Q}}_{L}J\right)\right)$;○Do for j○Calculate: ${\bar{Q}}_{L}=\sum_{k=1}^{p}{\hat{\sigma }}_{k}{\bar{Q}}_{Lk}$○Establish the cost function:$\underset{P_{a}^{U}}{\min}\left(\frac{1}{2}f{\left(P_{a}^{U}\right)}^{T}{\bar{Q}}_{L}f\left(P_{a}^{U}\right)\right)$, see Equations (9)–(11)○Update the Jacobian Matrix J:$J=\left[\begin{array}{ccc} \frac{\partial f_{1}\left(P_{a}^{U}\right)}{\partial x_{a}^{U}} & \frac{\partial f_{1}\left(P_{a}^{U}\right)}{\partial y_{a}^{U}} & \frac{\partial f_{1}\left(P_{a}^{U}\right)}{\partial z_{a}^{U}}\\ \frac{\partial f_{2}\left(P_{a}^{U}\right)}{\partial x_{a}^{U}} & \frac{\partial f_{2}\left(P_{a}^{U}\right)}{\partial y_{a}^{U}} & \frac{\partial f_{2}\left(P_{a}^{U}\right)}{\partial z_{a}^{U}}\\ \vdots & \vdots & \vdots \\ \frac{\partial f_{n}\left(P_{a}^{U}\right)}{\partial x_{a}^{U}} & \frac{\partial f_{n}\left(P_{a}^{U}\right)}{\partial y_{a}^{U}} & \frac{\partial f_{n}\left(P_{a}^{U}\right)}{\partial z_{a}^{U}} \end{array}\right]$ see Equation (12).○Calculate the $\Delta P_{a}^{U}$ and obtain $P_{a\left[j+1\right]}^{U}$ or $P_{a}^{U}$:$\;\;\;\;\left(J^{T}{\bar{Q}}_{L}J+\mu I\right)\Delta P_{a}^{U}=J^{T}{\bar{Q}}_{L}\left(\hat{f}\left(P_{a\left[j\right]}^{U}\right)\right)$◆if $\left\|\Delta P_{a}^{U}\right\|<\varepsilon_{0}$$\;\;\;P_{a}^{U}=P_{a\left[j\right]}^{U},\;\mathrm{then}\;\mathrm{end}\;\mathrm{do}\;\mathrm{over}\;j.$◆else⟡Calculate the damping adjustment factor ρ:   ρ=FPajU−FPaj+1UΔPaUμΔPaU+JTQ¯LfPajUT⟡if $\rho >\varepsilon_{1}$$\;\;\;P_{a\left[j+1\right]}^{U}=P_{a\left[j\right]}^{U}+\Delta P_{a}^{U}$$\;\;\;\mu =\mu \max\left(1/3,1-{\left(2\rho -1\right)}^{3}\right);\nu =2;$⟡else   μ=μv,v=2v;

●Calculate $P_{A}^{⊥}=I-A{\left(A^{T}Q_{L}^{-1}A\right)}^{-1}A^{T}Q_{L}^{-1}$●Calculate N and l according to Equations (16) and (17).●Increase i=i+1, then estimate:$\;\;\;\hat{\sigma }=\arg\underset{\sigma \geq 0}{\min}\left(\frac{1}{2}\sigma^{T}N\sigma -l^{T}\sigma \right)\;\mathrm{and}\;\mathrm{update}:\;{\hat{\sigma }}_{\left[i\right]}=\hat{\sigma }$●Repeat until $\left\|{\hat{\sigma }}_{\left[i\right]}-{\hat{\sigma }}_{\left[i-1\right]}\right\|<\varepsilon_{2}$●End do over i

In practical application scenarios, relative weight ratios between various types of observations are unreasonable if the observation covariance is inaccurate. To enhance the impact of this change on estimation accuracy, LS-VCE is integrated into the original model to construct an adaptive anchor position estimation algorithm, thereby reducing estimation errors.

Because only m2 and m3 used multiple observations, we integrate LS-VCE into m2 and m3 for constructing an adaptive anchor position estimation algorithm, represented by m2-LS-VCE and m3-LS-VCE, respectively.

### 4.3. Performance Evaluation of the Proposed Anchor Position Estimation Algorithm

In this section, the Cramer–Rao Lower Bound (CRLB) expression considering curvature effects is derived. The CRLB provides a lower bound for any unbiased estimator’s mean square error (MSE) of an unknown parameter. For the distance and angle model in Equations (3)–(5), the joint probability density function (pdf) of the measurement noise is

(19) $$p\left(\hat{d}|P_{a}^{U}\right)=\frac{1}{{\left(2\pi \right)}^{\frac{N}{2}}{\left|\sum_{d}\right|}^{\frac{1}{2}}}e^{\left\{-\frac{1}{2}{\left(\hat{d}-d\left(P_{a}^{U}\right)\right)}^{T}\sum_{d}^{-1}\left(\hat{d}-d\left(P_{a}^{U}\right)\right)\right\}}$$

(20) pd^,α^|PaU=12πNΣd12Σα12e−12d^−dPaUTΣdd^−dPaU−1−12α^−αPaUTΣαα^−αPaU−1

(21) pd^,α^,β^|PaU=12π3N2Σd12Σα12Σβ12eoo=−12d^−dPaUTΣdd^−dPaU−1−12α^−αPaUTΣαα^−αPaU−1−12β^−βPaUTΣββ^−βPaU−1

where $P_{a}^{U}={\left[\begin{array}{ccc} x_{a}^{U} & y_{a}^{U} & z_{a}^{U} \end{array}\right]}^{T}$, $\hat{d}={\left[{\hat{d}}_{1},{\hat{d}}_{2},\cdots ,{\hat{d}}_{N}\right]}^{T}$, $\hat{\alpha }={\left[{\hat{\alpha }}_{1},{\hat{\alpha }}_{2},\cdots ,{\hat{\alpha }}_{N}\right]}^{T}$, $\hat{\beta }={\left[{\hat{\beta }}_{1},{\hat{\beta }}_{2},\cdots ,{\hat{\beta }}_{N}\right]}^{T}$, $\Sigma_{d}^{-1}=diag\left\{\Omega_{d_{1}}^{-2},\Omega_{d_{2}}^{-2},\cdots ,\Omega_{d_{N}}^{-2}\right\}$.

Using Equation (19), the logarithmic likelihood function of the distance joint pdf can be written as

(22) $$\ln\left[p\left(\hat{d}|P_{a}^{U}\right)\right]=-\frac{1}{2}\sum_{k=1}^{N}\Omega_{d_{k}}^{-2}{\left[\hat{d}-d\left(P_{a}^{U}\right)\right]}_{k}^{2}-c_{k}$$

where ${\left[\;\right]}_{k}$ denotes the kth element of the corresponding vector and $c_{k}=\ln\left(2\pi \sigma_{d_{k}}^{2}\right)$. The FIM corresponding to Equations (19)–(21) can be derived, respectively, as follows:

(23) $$F_{d}\left(P_{a}^{U}\right)=\mathrm{cov}\left(\frac{\partial }{\partial P_{a}^{U}}\ln\left[p\left(\hat{d}|P_{a}^{U}\right)\right]\right)$$

(24) $$\begin{array}{c} F_{d,\alpha }\left(P_{a}^{U}\right)=-E\left(\frac{\partial^{2}\ln\left(p\left(\hat{d},\hat{\alpha }|P_{a}^{U}\right)\right)}{\partial P_{a}^{U}\partial P_{a}^{UT}}\right)\\ ={\left(\frac{\partial d}{\partial P_{a}^{UT}}\right)}^{T}\Sigma_{d}^{-1}\frac{\partial d}{\partial P_{a}^{UT}}+{\left(\frac{\partial \alpha }{\partial P_{a}^{UT}}\right)}^{T}\Sigma_{\alpha }^{-1}\frac{\partial \alpha }{\partial P_{a}^{UT}} \end{array}$$

(25) $$\begin{array}{c} F_{d,\alpha ,\beta }\left(P_{a}^{U}\right)=-E\left(\frac{\partial^{2}\ln\left(p\left(\hat{d},\hat{\alpha },\hat{\beta }|P_{a}^{U}\right)\right)}{\partial P_{a}^{U}\partial P_{a}^{UT}}\right)\\ ={\left(\frac{\partial d}{\partial P_{a}^{UT}}\right)}^{T}\Sigma_{d}^{-1}\frac{\partial d}{\partial P_{a}^{UT}}+{\left(\frac{\partial \alpha }{\partial P_{a}^{UT}}\right)}^{T}\Sigma_{\alpha }^{-1}\frac{\partial \alpha }{\partial P_{a}^{UT}}+{\left(\frac{\partial \beta }{\partial P_{a}^{UT}}\right)}^{T}\Sigma_{\beta }^{-1}\frac{\partial \beta }{\partial P_{a}^{UT}} \end{array}$$

Assume $F_{d}\left(P_{a}^{U}\right)$, $F_{d,\alpha }\left(P_{a}^{U}\right)$, $F_{d,\alpha ,\beta }\left(P_{a}^{U}\right)$ exists, the variance of any element $P_{a,n}^{U}$ of $P_{a}^{U}$ is bounded below by $F_{d}{\left(P_{a}^{U}\right)}_{nn}$, $F_{d,\alpha }{\left(P_{a}^{U}\right)}_{nn}$, $F_{d,\alpha ,\beta }{\left(P_{a}^{U}\right)}_{nn}$. The CRLB of the anchor position estimations for $F_{d}\left(P_{a}^{U}\right)$, $F_{d,\alpha }\left(P_{a}^{U}\right)$, $F_{d,\alpha ,\beta }\left(P_{a}^{U}\right)$ is

(26) $$\sigma_{P_{a}^{U},d}^{2}=tr\left[F_{d}^{-1}\left(P_{a}^{U}\right)\right]$$

(27) $$\sigma_{P_{a}^{U},d,\alpha }^{2}=tr\left[F_{d,\alpha }^{-1}\left(P_{a}^{U}\right)\right]$$

(28) $$\sigma_{P_{a}^{U},d,\alpha ,\beta }^{2}=tr\left[F_{d,\alpha ,\beta }^{-1}\left(P_{a}^{U}\right)\right]$$

where the Fisher Information Matrix (FIM) $F_{d}\left(P_{a}^{U}\right)$, $F_{d,\alpha }\left(P_{a}^{U}\right)$, $F_{d,\alpha ,\beta }\left(P_{a}^{U}\right)\in R^{3\times 3}$, $tr\left[\cdot \right]$ is the trace operation of covariance matrix. To investigate the relationship between trajectory curvature and anchor position estimation error, the trajectory is assumed to be a circular path with varying curvature. According to the polar equation of the arc, the equation for the curved trajectory is expressed as follows:

(29) $$\left\{\begin{array}{l} x=\frac{\cos\theta }{\rho_{c}}\\ y=\frac{\sin\theta }{\rho_{c}}+\frac{1}{\rho_{c}}\\ z=0 \end{array}\right.$$

where $\rho_{c}$ represents the curvature of trajectory, θ represents the angle corresponding to a point on the curved trajectory.

**Figure 2:**
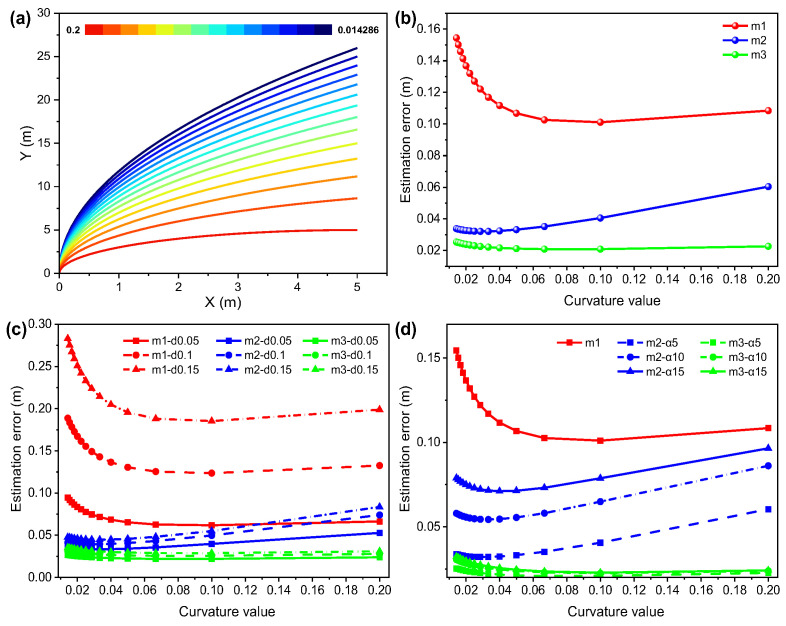
(**a**) The trajectories with different curvatures (inset: the range of color bar is consistent with curvature value from 0.2~0.014286); (**b**) the CRLB results of the three methods varying with curvature of trajectory under Gaussian noise $\Omega_{d}=0.1\;m,\Omega_{\alpha }=5^{\circ },\Omega_{\beta }=5^{\circ }$; (**c**) the CRLB results of the three methods varying in curvature of trajectory when $\Omega_{\alpha }=5^{\circ },\Omega_{\beta }=5^{\circ }$ and $\Omega_{d}$ is set to 0.05 m, 0.1 m, 0.15 m respectively, and (**d**) the CRLB results of the three methods varying in curvature of trajectory when $\Omega_{d}=0.1\;m,\Omega_{\beta }=5^{\circ }$ and $\Omega_{\alpha }$ is set to $5^{\circ }$, $10^{\circ }$, and $15^{\circ }$, respectively.

Figure 2a shows that the trajectories with different curvatures, Figure 2b–d are based on Figure 2a. Figure 2b illustrates the significant differences in estimation accuracy among the three algorithms (m1, m2, m3) under the same conditions, where m3 consistently yields the lowest error, m2 performs moderately, and m1 exhibits substantially larger errors in the low curvature region compared with the proposed two algorithms. This indicates that, for low-curvature trajectories, the proposed algorithms achieve more accurate anchor position estimation. Furthermore, Figure 2c,d investigate the effects of range error and azimuth error on the anchor estimation accuracy of the three algorithms. Figure 2c shows that the estimation accuracy of m2 and m3 is less affected than m1 according to range error changing, and Figure 2d shows that the estimation accuracy of m3 is also less affected even with azimuth error changing in the low curvature range (X < 0.04 m), which demonstrates that the proposed algorithm m3 is almost insensitive to changes in range error and azimuth error, and shows better robustness for estimation of anchor position in straight and small-curvature trajectories.

## 5. Experimental Results and Analysis

In this section, simulation experiments and real experiments are conducted to evaluate the feasibility of the proposed UWB anchor position estimation method for various conditions and also demonstrate the high precision of the proposed UWB anchor position estimation method. The data of simulation experiment is generated by Visual Studio Code 1.74.3 and MATLAB2022a. The data noise simulated is set according to a Gaussian distribution.

### 5.1. Simulation Experiment

**Figure 3:**
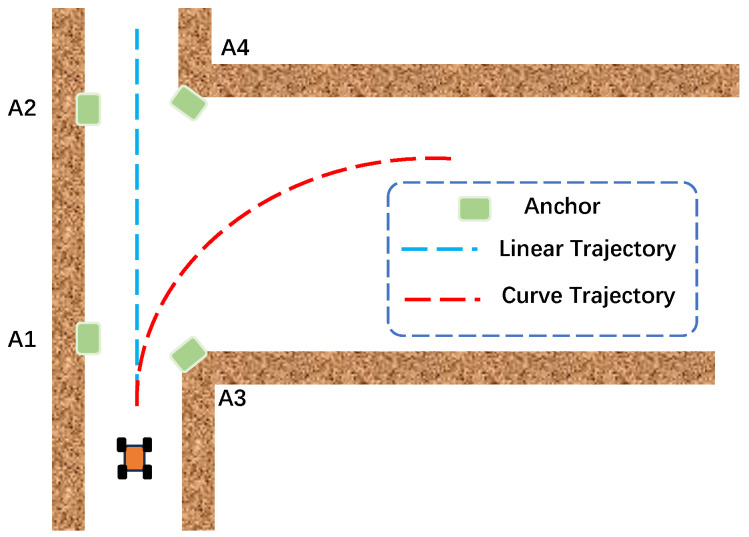
Simulated corridor environment and four anchors’ deployment.

The simulation environment is assumed to be a corridor, as shown in Figure 3. UWB anchors are installed on the walls on both sides. The previous literature [12] reports UWB anchor position estimation based on range measurement for trajectories with large curvature, but this estimation algorithm is not accurate for straight lines and small-curvature trajectories. Table 1 shows the simulated true value of four anchors’ positions.

The simulation experiments are categorized into the following two parts:

(1) The consistency of the UWB anchor position estimation algorithm is validated under fixed measurement variance.
(2) Investigate the estimation accuracy of the UWB anchor position algorithm under varying measurement variance.

#### 5.1.1. Consistency Simulation Experiment

To verify the consistency of m1, m2 and m3, range data is the Euclidean norm of ground truth positions with added Gaussian noise with standard deviation $\Omega_{d}=0.1\;m$. Azimuth and elevation angle are calculated by Equations (4) and (5) with added Gaussian noise with standard deviation $\Omega_{\alpha }=\Omega_{\beta }=5^{\circ }$. The simulated UWB data outputs at the same frequency (20 Hz) as the ground truth position data.

**Figure 4:**
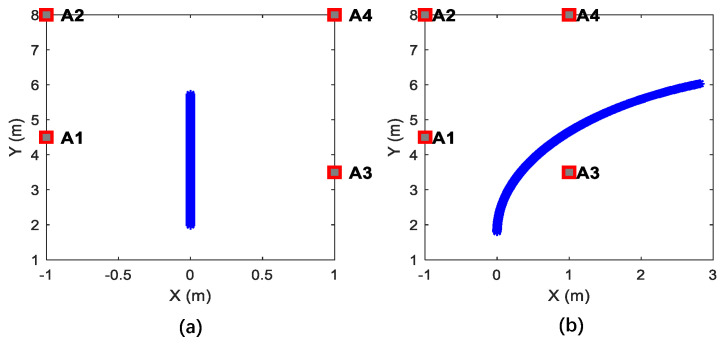
Simulated trajectories and UWB anchor deployment. (**a**) Straight trajectory, (**b**) curve motion trajectory.

UWB anchor deployment and the simulated trajectories are shown in Figure 4. Based on the predefined multi-observation error parameters, 10 sets of observation data were systematically generated for simulated trajectories.

**Figure 5:**
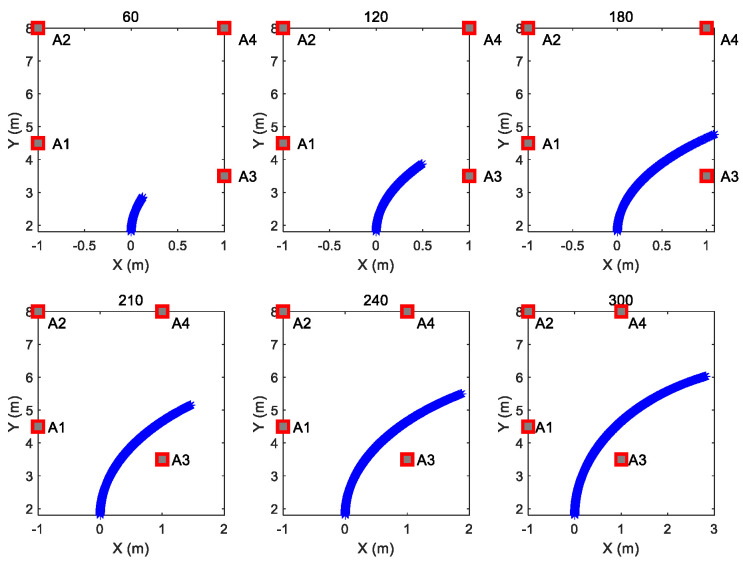
Corresponding trajectory curves of 6 observation numbers. The 6 observation numbers are 60, 120, 180, 210, 240 and 300, respectively.

In order to compare the convergence speed of the three algorithms, six observation numbers were selected from the trajectory. Figure 5 shows the corresponding parts of the trajectory of the selected six observation numbers in Figure 4b curve trajectory as an example.

**Figure 6:**
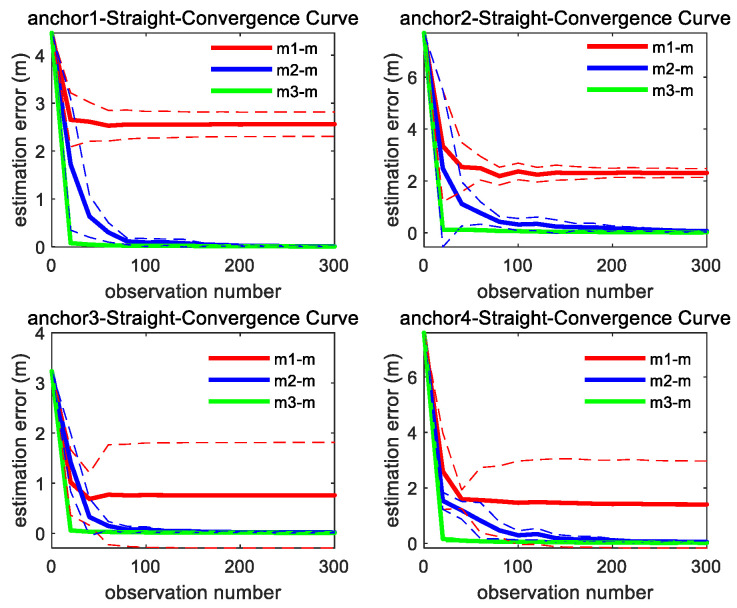
Convergence curve of four anchors’ position estimation under straight trajectory; the solid line represents the mean of the 10 sets of data estimation results, and the same color dashed line represents the corresponding variance fluctuation.

As shown in Figure 6, under the linear motion trajectory scenario, m3 achieves the fastest convergence rate, followed by m2. Although the estimation error of m1 reduces at the beginning stage, it ultimately retains a significant residual error.

As presented in Table 2, the linear trajectory of UGV aligned along the Y-axis imposes only directional constraints in the Y-dimension, which assures the Y-coordinate estimation by m1 based on range to converge toward the simulation-specified ground truth, while X-coordinate and Z-coordinate estimation by m1 cannot converge toward the simulation-specified ground truth sufficiently. By introducing cross-axis constraints between X and Y, m2 enhances the estimation precision of the X-coordinate and Z-coordinate, while m3 further incorporates three-dimensional constraints across X, Y, and Z coordinates and achieves more estimation precision than m2.

**Figure 7:**
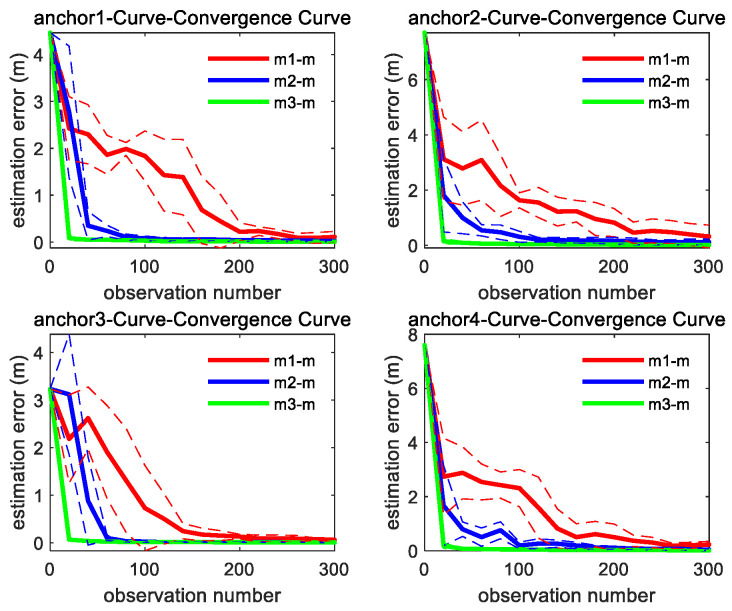
Convergence curve of four anchors’ position estimation under curve motion trajectory; the solid line represents the mean of the 10 sets of data estimation results, and the same color dashed line represents the corresponding variance fluctuation results.

As shown in Figure 7, the convergence speed order of the three methods is the same as straight trajectory. It can be found from Figure 6 and Figure 7 that through integrating the azimuth angle to m1, m2 imposes a constraint between the X and Y coordinate, which makes the estimation convergence speed of m2 better than m1, while m3 further integrates the elevation angle to m2, which maximally restricts the solution space for unknown anchor locations and makes m3 achieve the best optimal convergence speed.

As evidenced in Table 3, it can be found that the four anchors’ position estimation accuracy of the three methods is very close, which indicates that the three methods have no significant difference when the UGV executes the big-curvature trajectory prescribed in Figure 4b. Inspired by the results shown in Table 3, distinct UGV motion curvature trajectories are designed to further evaluate the difference in the three methods on anchor position estimation accuracy.

**Figure 8:**
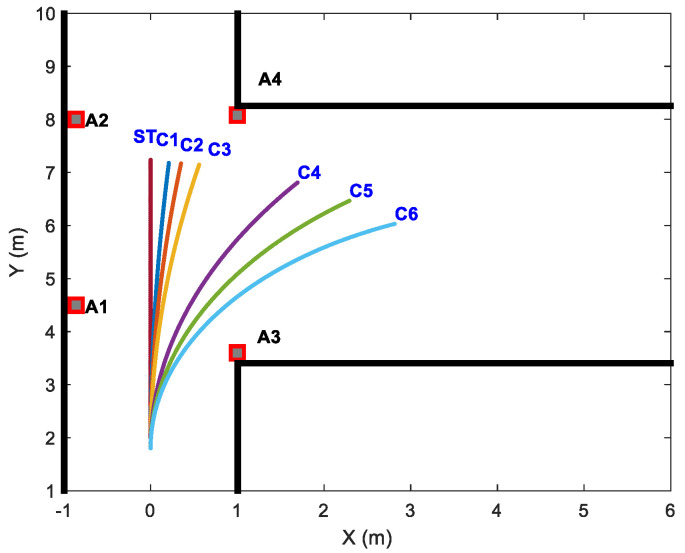
Simulated straight and curved trajectories of different curvatures.

Experiment trajectories with different curvatures are designed as follows:

Based on the predefined multi-observation error parameters, 10 sets of observation data were systematically generated for each curve trajectory in Figure 8 to rigorously validate the algorithm’s consistency. The curvatures of simulated curve trajectories (C1–C6) are presented in Table 4.

**Figure 9:**
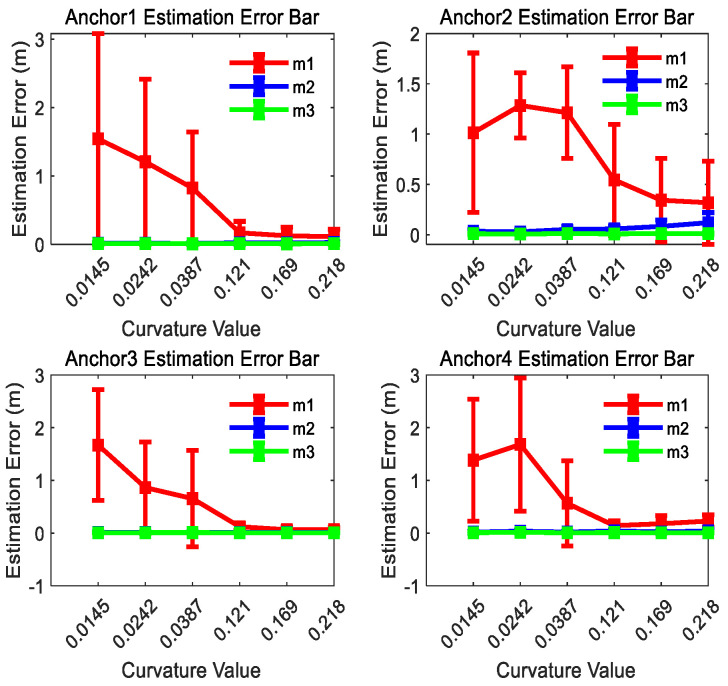
UWB anchor position estimation error bar result comparison of three methods for curve trajectories with different curvatures at the 300th observation number.

The estimated position results for anchors 1, 2, 3, and 4 are shown in Figure 9 for comparison. As demonstrated in Figure 9, the position estimation error of m1 exhibits an inverse relationship with trajectory curvature: the higher the curvature of the motion trajectory is, the smaller the position estimation error will be. If the curvature of the motion trajectory is big enough, m1 attains comparable positioning accuracy to m2 and m3. It is worth noting that the estimation performance of m2 and m3 is less affected by curvature changes. This result proves that the anchor position estimation method proposed in this paper improves the position estimation accuracy effectively for linear or small-curvature trajectories.

Figure 9 also shows that the dispersion of the estimation results of m1 is the biggest in the three methods for each curvature trajectory, and the dispersion degree of m1 is inverse with the curvature of the curve trajectories. It can be concluded that the estimation results of m2 and m3 have smaller dispersion and better consistency than m1 for each curvature trajectory.

#### 5.1.2. Adaptive Nonlinear Optimization Anchor Position Estimation Method Accuracy Verification Simulation Experiment

To further validate the accuracy of the proposed algorithms in the paper when outliers appear, based on the predefined multi-observations error parameters, 10 sets of observation data were systematically generated for each trajectory in Figure 8. In addition, 60 outliers whose magnitudes are uniformly distributed from 3 to 10 times the corresponding prior standard deviations are generated and added to the original observations with random errors.

After the outliers are added, the following five computational schemes are employed to compare their robustness.

**Scheme 1:** Nonlinear optimization anchor position estimation method based on cost function (9), i.e., m1. The standard deviation of range is $\Omega_{d}=0.1\;m$.

**Scheme 2:** Nonlinear optimization anchor position estimation method based on cost function (10), i.e., m2. The standard deviation of range and azimuth is $\Omega_{d}=0.1\;m$, $\Omega_{\alpha }=5^{\circ }$.

**Scheme 3:** Nonlinear optimization anchor position estimation method based on cost function (11), i.e., m3. The standard deviation of range, azimuth, and elevation angle is $\Omega_{d}=0.1\;m$, $\Omega_{\alpha }=5^{\circ }$, $\Omega_{\beta }=5^{\circ }$.

**Scheme 4:** Adaptive nonlinear optimization anchor position estimation method integrating cost function (10) and LS-VCE algorithm, i.e., m2-LS-VCE.

**Scheme 5:** Adaptive nonlinear optimization anchor position estimation method integrating cost function (11) and LS-VCE algorithm, i.e., m3-LS-VCE.

In Scheme 4, the initial values of the variance components corresponding to the two classes of original observations $\left(d,\alpha \right)$ are set as $\sigma_{d}=1,\sigma_{\alpha }=1$.

In Scheme 5, the initial values of the variance components corresponding to the three classes of original observations $\left(d,\alpha ,\beta \right)$ are set as $\sigma_{d}=1,\sigma_{\alpha }=1,\sigma_{\beta }=1$.

**Figure 10:**
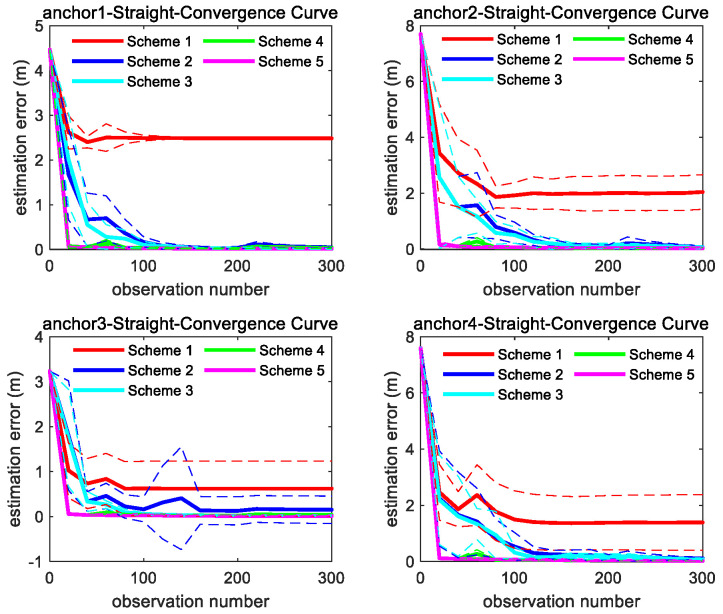
Convergence curve of four anchors’ position estimation under straight trajectory in Figure 8; the solid line represents the mean of the 10 sets of data estimation results, and the same color dashed line represents the corresponding variance fluctuation of the 10 sets of data estimation results.

**Figure 11:**
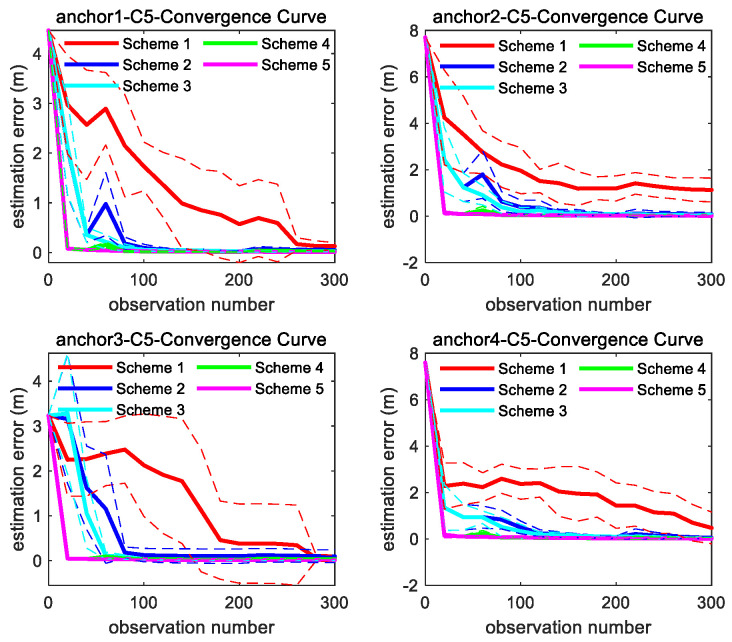
Convergence curve of four anchors’ position estimation under C5 trajectory in Figure 8; the solid line represents the mean of the 10 sets of data estimation results, and the same color dashed line represents the corresponding variance fluctuation of the 10 sets of data estimation results.

**Figure 12:**
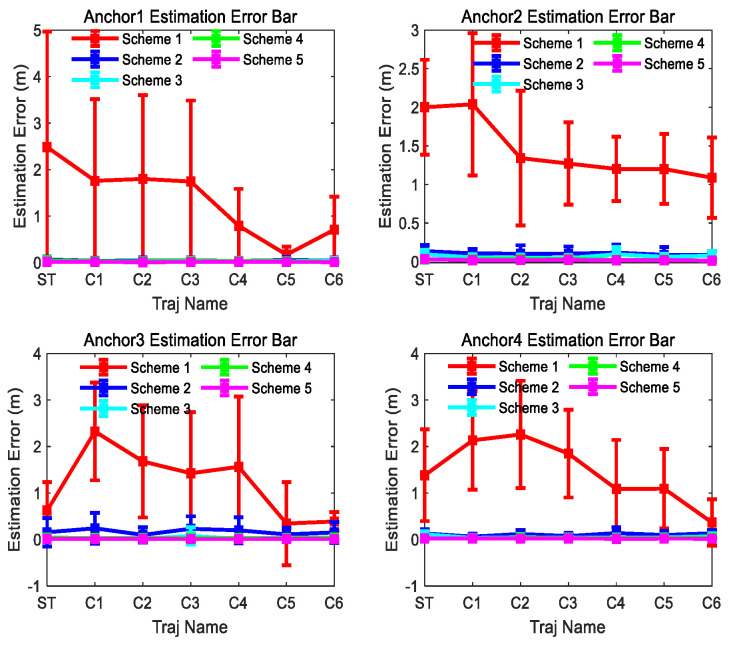
Scheme 1~5 anchor estimation error bar results of the 300th observation number for different trajectories in Figure 8.

**Figure 13:**
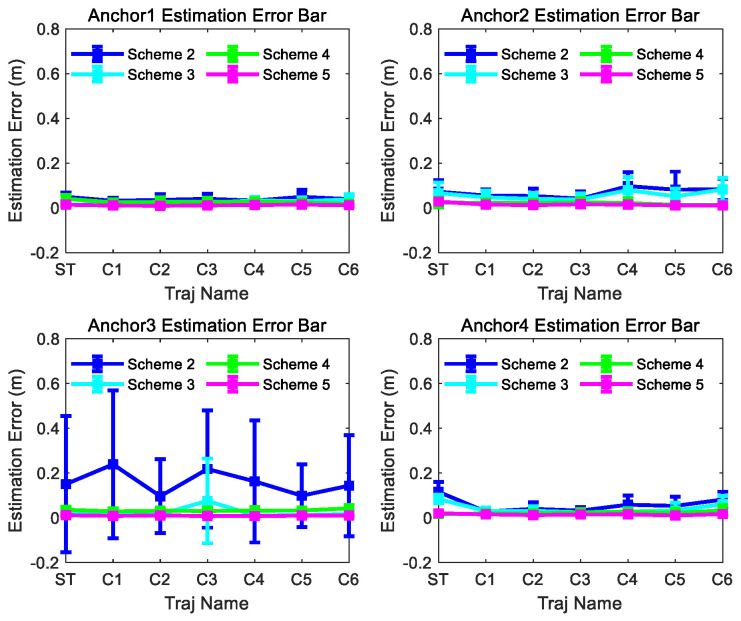
Scheme 2~5 anchor estimation error bar results of the 300th observation number for different trajectories in Figure 8.

Figure 10 and Figure 11 show the consistency and accuracy of the 5 Schemes simulation results for trajectory ST and C5 in Figure 8, respectively. Figure 12 and Figure 13 show the consistency and accuracy of the 5 Schemes simulation results for different curvature trajectories. In order to clearly display the estimation error bar results of Scheme 2~5, Figure 13 removes the Scheme 1 estimation error bar results.

Based on the results displayed in Figure 9, Figure 10, Figure 11, Figure 12 and Figure 13, we can find the following points.

(1) Even though the outliers are not added in the observations, Scheme 1 fails to achieve ideal anchor position estimation results for trajectories C1–C3 in Figure 8. It is also found that significant fluctuations exist in the estimation error results obtained using Scheme 1.
(2) Comparing with the results of Scheme 1, Scheme 2 can obtain more stable and satisfactory results, and the results of Scheme 2 under different trajectories are almost unaffected by the trajectory. But after adding the outliers, the anchor position estimation accuracy of Scheme 2 decreases. The estimation results of Scheme 4, integrating the LS-VCE and Scheme 2, demonstrate superior consistency and accuracy compared to those of Scheme 2.
(3) Comparing with the anchor estimation accuracy of Scheme 1 and Scheme 2, the anchor estimation accuracy of Scheme 3 is the highest before adding the outliers into the three types of observations. But, after adding the outliers, the estimation accuracy of Scheme 3 is inferior to that of Scheme 4 under certain trajectories. After Scheme 5 integrates the LS-VCE and Scheme 3, the consistency and accuracy of Scheme 5 outper-form Scheme 3 and Scheme 4.

The simulation results show that after integrating LS-VCE, the method proposed in this paper has stronger robustness for various trajectories, whether outliers appear or not.

### 5.2. Real Experiments

**Figure 14:**
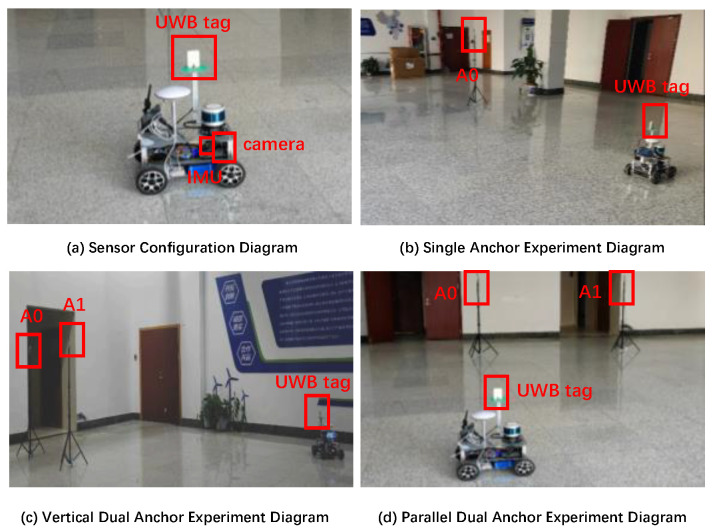
Digital photos of experimental equipment.

To validate the accuracy of the proposed anchor position estimation algorithm in practical scenarios, we conducted real ground vehicle experiments encompassing single-anchor and dual-anchor localization with unknown position. In order to evaluate the possible influence of anchor deployment on estimation results, the dual-anchor configurations are in two distinct geometric arrangements, i.e., one is side by side or parallel, the other is front and back or vertical. The experimental setup details are presented in Figure 14.

The UWB sensor module installed on the UGV in the experiment integrates DecaWave’s official DW32XX as the core UWB chip, equipped with an onboard dual-antenna system for angle measurement, IMU, and a camera. Its key specifications are summarized in Table 5.

Due to the lack of antenna array equipment, the elevation angle cannot be calculated, making it impossible to verify the accuracy of the m3 and m3-LS-VCE algorithms in practical scenarios. So, in the subsequent practical experiments, only the validation of the estimation accuracy of m1, m2, and m2-LS-VCE algorithms will be involved.

**Figure 15:**
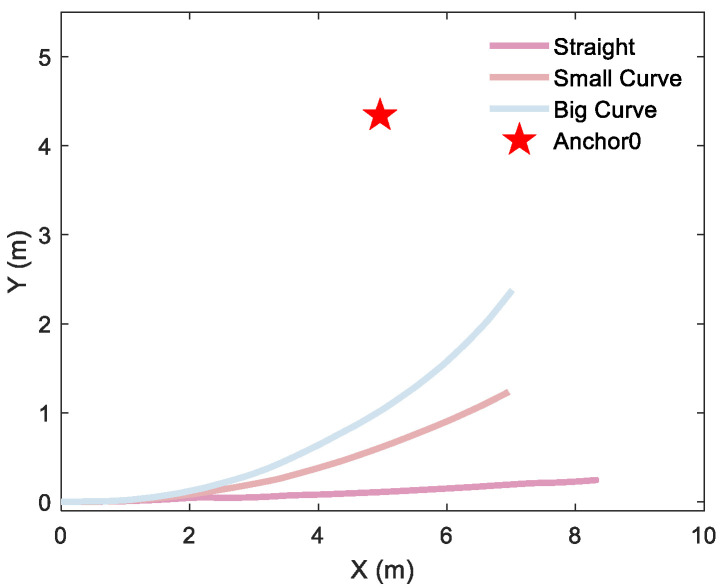
Different motion trajectories of a single anchor: a 2D straight trajectory and a 2D curve trajectory with a curvature radius of 19 m and 10 m, respectively.

**Figure 16:**
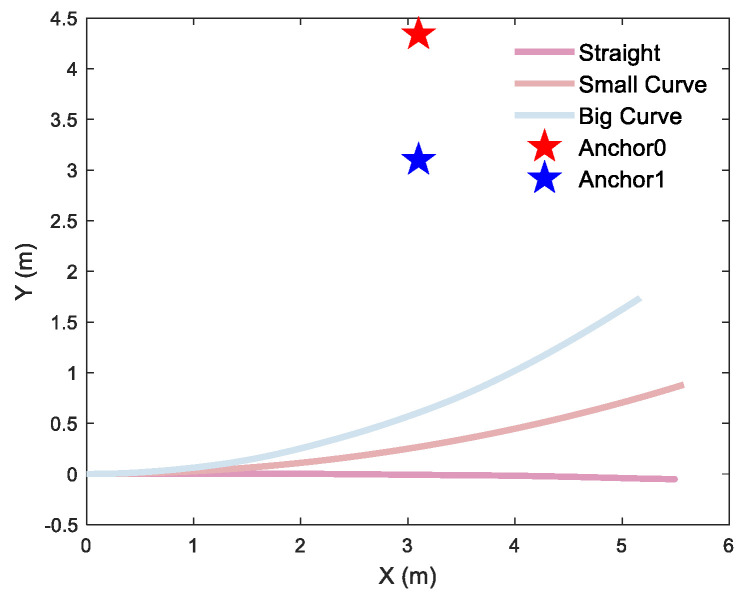
Different trajectories of dual-anchor under vertical deployment: a 2D straight trajectory and 2D curve trajectory with a curvature radius of 18 m and 8.8 m, respectively.

**Figure 17:**
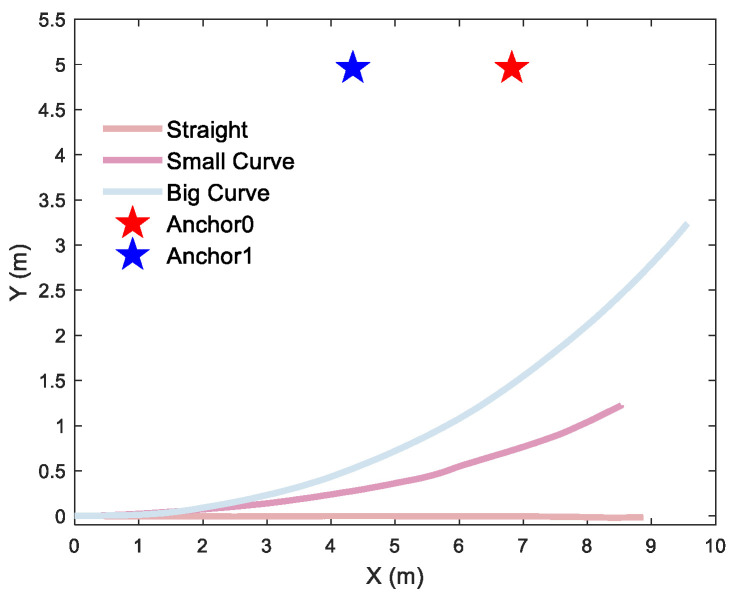
Different trajectories of dual-anchor under parallel deployment: a 2D straight trajectory and a 2D curve trajectory with a curvature radius of 27 m and 14 m, respectively.

To quantify the curvature-dependent performance of the localization algorithm, rigorous testing was conducted with a differential-driven UGV traversing three distinct curvature trajectories for single-anchor and dual-anchor deployment, respectively. Single-anchor measured trajectories are shown in Figure 15, dual-anchor measured trajectories of vertical and parallel deployment are shown in Figure 16 and Figure 17, respectively.

**Figure 18:**
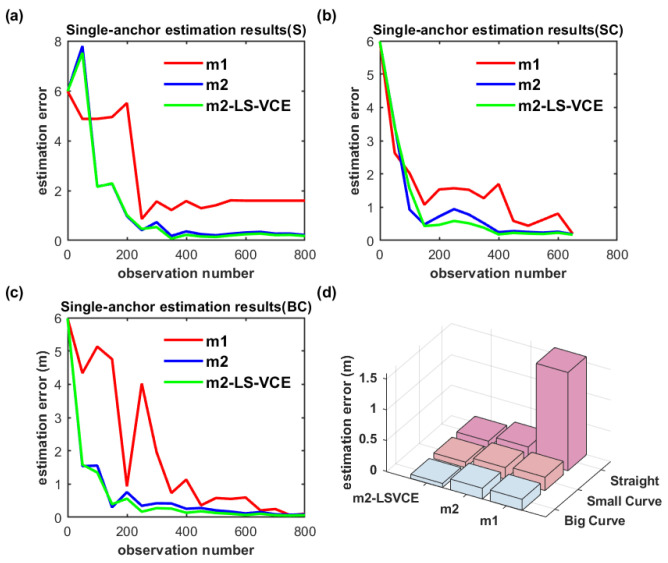
(**a**–**c**) Convergence curve of single-anchor position estimation for three distinct trajectories, and (**d**) single-UWB-anchor position estimation results of three methods for distinct trajectories.

In Figure 18a–c, S, SC, and BC denote straight, small-curvature, and big-curvature trajectories, respectively; the same denotation is used in the following figure. The UWB anchor position estimation results for the single-anchor setup with m1, m2, m2-LS-VCE methods under practical scenarios are illustrated in Figure 18d. The convergence speed of m2-LS-VCE is the fastest among the three methods, and the convergence speed of m2 is faster than that of m1, as shown in Figure 18a–c. As for the UWB anchor position estimation error, m2-LS-VCE method is also the best of the three methods. Compared to the position estimation error of m2 for straight, small-curvature, and big-curvature trajectories, the UWB anchor position estimation error of m2-LS-VCE has been reduced by 46.3%, 52.4%, and 62.3%, respectively, as shown in Figure 18d.

**Figure 19:**
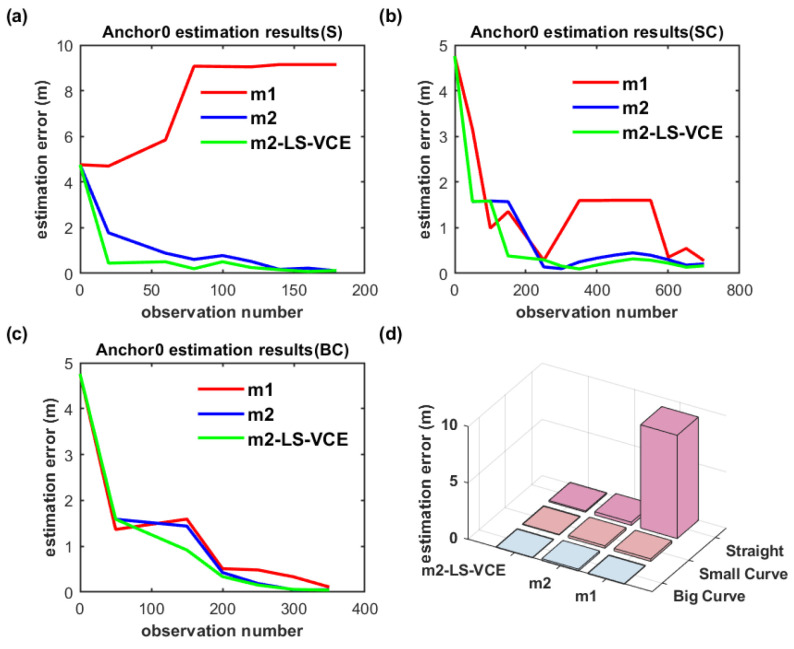
(**a**–**c**) Convergence curve of Anchor0 position estimation under dual-anchor vertical deployment for three distinct trajectories, and (**d**) Anchor0 position estimation results with three methods for three distinct trajectories under vertical deployment.

**Figure 20:**
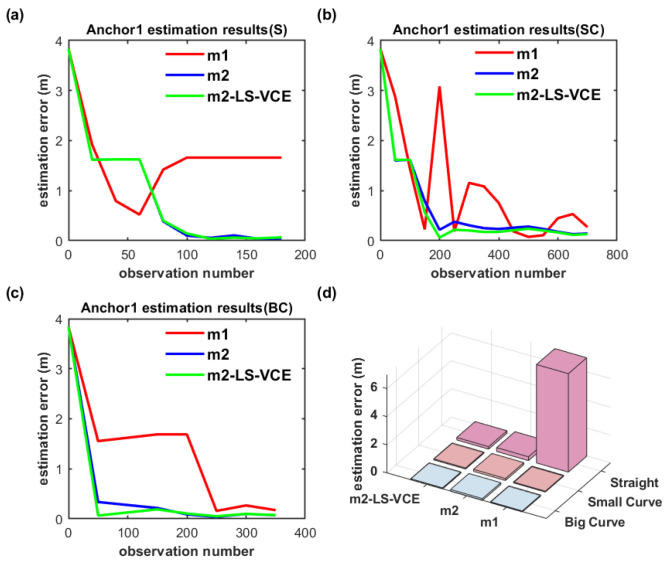
(**a**–**c**) Convergence curve of Anchor1 position estimation under dual-anchor vertical deployment for three distinct trajectories, and (**d**) Anchor1 position estimation results with three methods for three distinct trajectories under vertical deployment.

The anchor position estimation results for the dual-anchor setup with m1, m2 and m2-LS-VCE methods under vertical deployment are illustrated in Figure 19 and Figure 20. Anchor0 position estimation results are shown in Figure 19, while Anchor1 position estimation results are shown in Figure 20. Anchor0 is deployed behind Anchor1 as shown in Figure 14. Whether in Figure 19a–c or in Figure 20a–c, the convergence speed and anchor position estimation accuracy results of the three methods are consistent with that of single-anchor deployment.

The position estimation accuracy of Anchor1 here is higher than that of Anchor0; the cause may be that Anchor1 is placed in front of Anchor0 and interferes with Anchor0.

**Figure 21:**
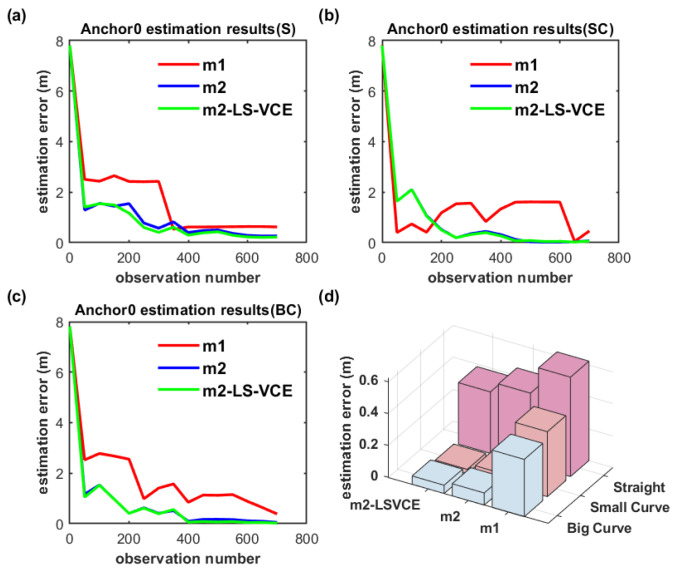
(**a**–**c**) Convergence curve of Anchor0 position estimation under parallel deployment for three distinct trajectories, and (**d**) Anchor0 position estimation results with three methods for three distinct trajectories under parallel deployment.

**Figure 22:**
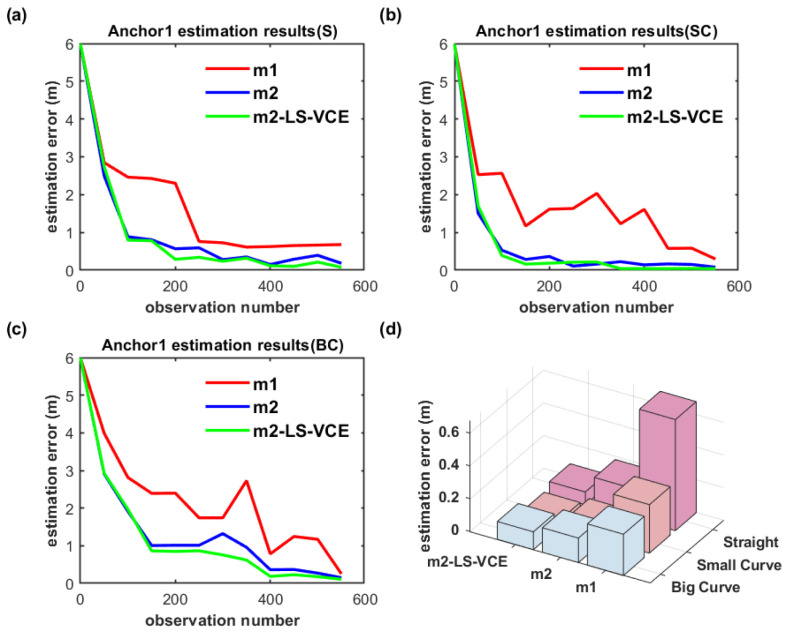
(**a**–**c**) Convergence curve of Anchor1 position estimation under parallel deployment for three distinct trajectories, and (**d**) Anchor1 position estimation results with three methods for three distinct trajectories under parallel deployment.

To further evaluate the position estimation method proposed in this paper, the dual-anchor setup under parallel deployment is also conducted. The position estimation results of anchors are illustrated in Figure 21 and Figure 22.

As shown in Figure 21a–c and Figure 22a–c, on the convergence speed and position estimation accuracy of the three methods, similar results to the dual-anchor vertical deployment experiment are achieved. Comparing to m2, the position estimation accuracy of Anchor0 using m2-LS-VCE for straight, small-curvature, and big-curvature trajectories under parallel deployment has been improved by 14.4%, 59.2%, and 31.6%, respectively, as shown in Figure 21d, and the position estimation accuracy of Anchor1 using m2-LS-VCE for straight, small-curvature and big-curvature trajectories under parallel deployment has been improved by 57.4%, 49.5%, and 27.4%, respectively, as shown in Figure 22d.

Due to the longer distance between Anchor0 and Anchor1, the electromagnetic signal interference between the two anchors is relatively smaller. Therefore, the position estimation accuracy of Anchor0 under parallel deployment is higher than that under vertical deployment.

Whether from simulation or real experiments, the anchor position estimation accuracy by the method proposed in this paper outdoes the method before. It is also found that, according to the results obtained from the above real experiments, the noise characteristics of multi-observation measurements are not constant and can be affected by environmental factors, leading to changes in covariance. Therefore, by integrating the variance component estimation algorithm into m2 (i.e., m2-LS-VCE) to reasonably modify the weights among multiple observations, the estimation accuracy can be made more accurate. It is also found that the anchor deployment can affect estimation results if the distance between anchors is relatively small, which may be worth researching further.

## 6. Conclusions

In this paper, a UWB anchor position estimation algorithm integrating range/azimuth and range/azimuth/elevation measurements is proposed. The consistency and accuracy of the algorithm are demonstrated in simulation and real experiments for various curvature trajectories. Because of integrating range/azimuth and range/azimuth/elevation measurements, the algorithm achieves higher anchor position estimation accuracy, faster convergence speed, and better consistency results in linear and small-curvature trajectories than that of the m1 method, i.e., based solely on the ranging residual model within the algorithmic framework of this paper.

To further enhance the robustness of the method, an adaptive weighting method (LS-VCE) has been integrated into the proposed algorithm in this paper for balancing the weights of multiple observables. Simulation experiments show stronger robustness for various curvature trajectories, whether outliers appear or not. Applying these to UGV, real experiment results also demonstrate better position estimation accuracy and convergence speed of the proposed algorithm under straight and small-curvature trajectory conditions, while maintaining a comparable estimation performance in big curvature scenarios. Furthermore, direct comparative analysis with other advanced algorithms in the literature will be conducted in future research.

## Figures and Tables

**Table 1:** The simulated anchors’ positions.

Anchor Num	Simulated Anchor Position		
	X (m)	Y (m)	Z (m)
A1	−1	4.5	1.8
A2	−1	8	1.4
A3	1	3.5	1.6
A4	1	8	1.6

**Table 2:** Four anchors’ position estimation results of linear trajectory.

Anchor	Method	Anchor Position Estimation		
		X (m)	Y (m)	Z (m)
A1	True	−1	4.5	1.8
	m1	1.467	4.504	1.442
	m2	−0.986	4.547	1.717
	m3	−1.009	4.501	1.796
A2	True	−1	8	1.4
	m1	1.588	7.997	0.698
	m2	−1.015	8.005	1.387
	m3	−1.018	8.003	1.412
A3	True	1	3.5	1.6
	m1	1.268	3.517	1.360
	m2	0.980	3.516	1.580
	m3	0.979	3.496	1.590
A4	True	1	8	1.6
	m1	1.688	7.992	0.838
	m2	0.977	7.982	1.639
	m3	1.014	7.985	1.610

**Table 3:** Four anchors’ position estimation results of curve trajectory.

Anchor	Method	Anchor Position Estimation		
		X (m)	Y (m)	Z (m)
A1	True	−1	4.5	1.8
	m1	−1.099	4.569	1.747
	m2	−1.094	4.565	1.750
	m3	−1.010	4.511	1.783
A2	True	−1	8	1.4
	m1	−0.985	8.030	1.421
	m2	−0.994	8.024	1.382
	m3	−0.995	8.015	1.378
A3	True	1	3.5	1.6
	m1	0.915	3.555	1.692
	m2	0.959	3.525	1.654
	m3	0.981	3.511	1.621
A4	True	1	8	1.6
	m1	1.015	7.963	1.649
	m2	1.027	7.969	1.635
	m3	1.005	7.981	1.619

**Table 4:** The curvature of simulated trajectories.

Curve Num	Curvature Value	Curve Num	Curvature Value
C1	0.0145	C4	0.1212
C2	0.0242	C5	0.1696
C3	0.0388	C6	0.2181

**Table 5:** UWB sensor module key parameters.

Parameter	Value/Range
Ranging Accuracy (1σ)	5 cm
Ranging Update Rate	Up to 100 Hz
Communication Data Rate	6.8 Mbps/27.2 Mbps/31.2 Mbps
Angle Accuracy (1σ)	8° (LOS)
Dynamic Range	±60°

## Data Availability

The original contributions presented in this study are included in the article. Further inquiries can be directed to the corresponding authors.
